# Development and outcomes of a primary care-based sleep assessment service in Canterbury, New Zealand

**DOI:** 10.1038/s41533-017-0030-1

**Published:** 2017-04-19

**Authors:** Michael J. Epton, Paul T. Kelly, Brett I. Shand, Sallyanne V. Powell, Judith N. Jones, Graham R. B. McGeoch, Michael C. Hlavac

**Affiliations:** 1 Sleep Unit, Department of Respiratory Medicine, Christchurch Hospital, Christchurch, New Zealand; 2 Canterbury Clinical Network, Canterbury District Health Board Christchurch, Christchurch, New Zealand; 3 The Canterbury Initiative, Canterbury District Health Board, Christchurch, New Zealand

## Abstract

Prior to 2007, increasing demand for sleep services, plus inability to adequately triage severity, led to long delays in sleep assessment and accessing continuous positive airway pressure. We established a community sleep assessment service carried out by trained general practices using a standardised tool and overnight oximetry. All cases were discussed at a multi-disciplinary meeting, with four outcomes: severe obstructive sleep apnoea treated with continuous positive airway pressure; investigation with more complex studies; sleep physician appointment; no or non-severe sleep disorder for general practitioner management. Assessment numbers increased steadily (~400 in 2007 vs. 1400 in 2015). Median time from referral to assessment and multi-disciplinary meeting was 28 and 48 days, respectively. After the first multi-disciplinary meeting, 23% of cases were assessed as having severe obstructive sleep apnoea. More complex studies (mostly flow based) were required in 49% of patients, identifying severe obstructive sleep apnoea in a further 13%. Thirty-seven percent of patients had obstructive sleep apnoea severe enough to qualify for funded treatment. Forty-eight percent of patients received a definitive answer from the first multi-disciplinary meeting. Median time from referral to continuous positive airway pressure for ‘at risk’ patients with severe obstructive sleep apnoea, e.g., commercial drivers, was 49 days, while patients with severe obstructive sleep apnoea but not ‘at risk’ waited 261 days for continuous positive airway pressure. Ten percent of patients required polysomnography, and 4% saw a sleep specialist. In conclusion, establishment of a community sleep assessment service and sleep multi-disciplinary meeting led to significantly more assessments, with short waiting times for treatment, especially in high-risk patients with severe obstructive sleep apnoea. Most patients can be assessed without more complex studies or face-to-face review by a sleep specialist.

## Sleep disorders: More assessments, shorter waits with community sleep service

A community-based service for common sleep disorders can provide rapid and easily accessed sleep assessment and treatment. A team led by Michael Hlavac and Michael Epton from Christchurch Hospital describe the creation of a sleep assessment service within the Canterbury district of New Zealand, in which initial assessments are conducted throughout the community by general practice teams under guidance and advice from sleep specialists at the region’s largest hospital. Before the service, there were around 300 sleep assessments per year in all of Canterbury, a region with a population of around 510,000. Now, that number has more than tripled, with shorter waiting times for treatment, especially for people with severe sleep apnoea. The authors conclude that most patients can be assessed for a suspected sleep disorder without needing to visit a hospital’s sleep unit.

## Introduction

Because of the increasing prevalence and recognition of obstructive sleep apnoea (OSA)^1^ and the increasing pressure on sleep specialty services in our setting, it is not practically or economically sustainable to assess all patients using traditional approaches such as sleep specialist assessment and full polysomnography. It is necessary to focus funded assessment processes to rapidly identify (1) symptomatic patients most at risk of adverse outcomes, including road traffic accidents, (2) symptomatic patients who require further more comprehensive assessment; and (3) patients who are minimally symptomatic or do not have a primary sleep disorder, to prevent excessive and inappropriate investigation and treatment. Mansfield and co-workers^2^ have recently published a review highlighting these issues and some possible solutions. There is also an increasing body of literature establishing the utility, cost efficiency and safety of a simplified and often community-based approach to the diagnosis and management of patients with suspected OSA.^3–5^ To reflect this evidence and increasing adoption of such changes in practice, the Australasian Sleep Association has recently developed guidelines that incorporate an estimation of pre-test probability and the use of simpler sleep studies as part of an overall OSA diagnostic algorithm.^6^

The Canterbury region has a population of 510,000, with 436,000 people living in greater Christchurch city.^7^ Previous surveys have established high levels of undiagnosed sleep disorders in the New Zealand population.^8^ Prior to 2007, the availability of specialised sleep services in Christchurch was extremely limited, with capacity to assess ~300 patients per year. Ability to triage referrals for severity was also limited due to inadequate referral information, resulting in patients with severe disease often waiting considerable times for assessment and treatment (up to 36 months from referral to continuous positive airway pressure (CPAP) treatment). Between May 2007 and April 2008, prior to the establishment of the new service, all referrals were managed by the hospital-based specialist sleep unit. A total of over 700 referrals were received over this time period. Of these 430 were seen by a sleep specialist. In 2007, the Ministry of Health required District Health Boards (DHBs) to provide specialist outpatient appointments within 6 months from referral. This lead to the establishment of a nurse assessment clinic, which managed 125 patients in the 12-month period from Jan 2008. Further patients were removed from the waiting list without any assessment.

In 2007, as part of the development of an integrated health system,^9^ known as the Canterbury Initiative, http://www.canterburyinitiative.org.nz/, primary and secondary care clinicians and health-care managers in Canterbury identified the need for a coordinated community-based assessment process for common sleep disorders such as OSA, based on the principles articulated above.

This paper describes the process of setting-up a community based service, with initial assessment being undertaken in general practice, linked to a multi-disciplinary meeting (MDM) process and specialist sleep diagnostics and treatment based out of Christchurch Hospital. The primary objectives of the service were to provide rapid and easily accessed sleep assessment within the community. The proposal was consistent with the ‘better, sooner, more convenient’ patient-focussed model proposed by the New Zealand government,^10^ and was able to build on already existing infrastructure and skills within general practice. Primary and secondary care clinicians, sleep physiologists and senior health-care management in Canterbury worked together to develop a sleep assessment service delivered predominantly in general practice, with an agreed assessment quality framework supported by Respiratory Specialist Services, and the hospital sleep service. Implementation of the service was supported by a training programme and a sleep disordered breathing (SDB) pathway on a locally developed clinical guidance website called Community HealthPathways^11–13^ www.cdhb.health.nz/Hospitals-Services/Health-Professionals/.../Health-Pathways.aspx. This upskilling of general practitioners (GPs) in the diagnosis and management of sleep-related breathing disorders was expected to reduce the number as well as improve the accuracy of referrals for specialist sleep assessments.

## Results

### Referrals and assessments

**Fig. 1:**
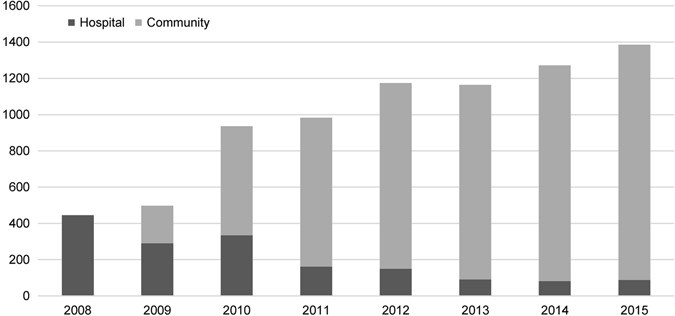
Changes in number and location of assessment of patients with sleep disordered breathing

Since the establishment of the community sleep assessment service in mid-2009, 22 of the 137 practices in the Canterbury region have become approved providers. There has been a steady increase in the number of assessments carried out in primary care between 2008 and 2015 (Fig. 1). The number of assessments at the hospital sleep unit has declined over the same time period from 445 in 2008 to 88 in 2015. The service delivered 6530 sleep assessments over this 6-year period. The ethnicity of the patients was similar to that described in the 2013 New Zealand census and included New Zealand European 84%, Mãori 7%, Pacific Islander 3%, Asian 3%, Middle Eastern/Latin American/African 1%, and not stated 2%.

### MDM and patient outcomes

**Fig. 2:**
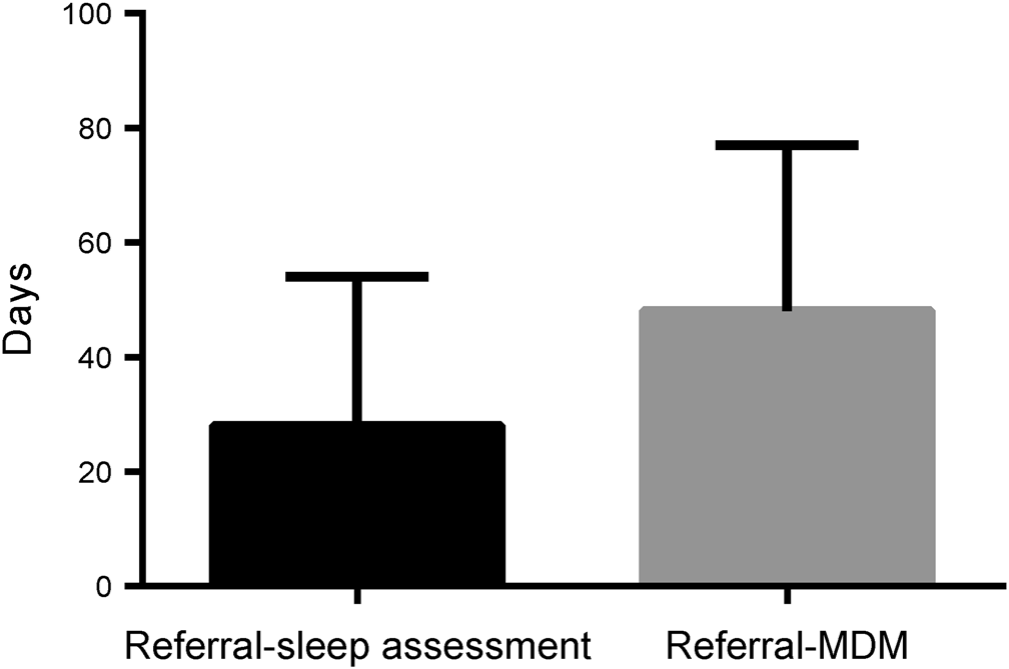
Wait-times (median and interquartile range) from referral to sleep assessment and MDM in 2014

Figure 2 shows the times from referral to assessment, and subsequent MDM discussion. 96 patients (8%) had a significant (>100 days) delay between referral and assessment.

**Fig. 3:**
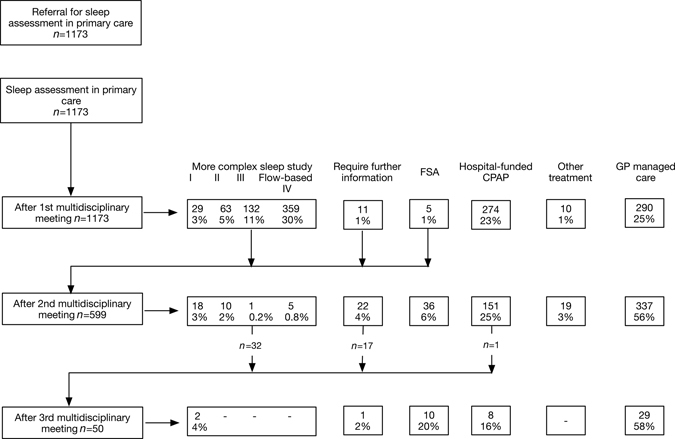
The numbers of patients flowing through the community assessment, MDM and treatment pathway during an example year, 2014

**Fig. 4:**
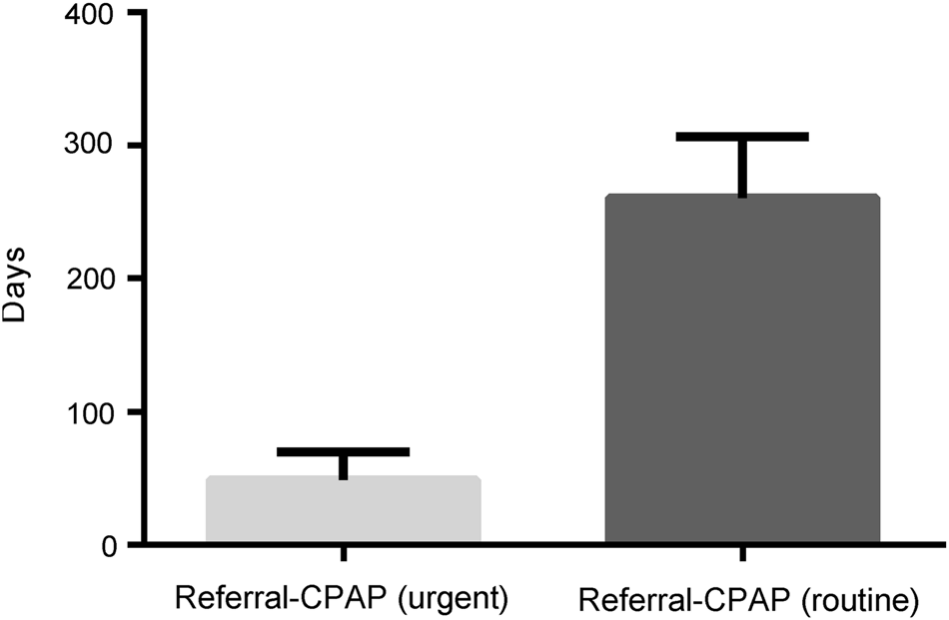
Wait-times from referral to sleep assessment, MDM discussion and CPAP therapy in 2014

The pathways and numbers for patients flowing through the community assessment, MDM and treatment pathway during an example year, 2014 are shown in Fig. 3. All data described in the following paragraphs of this section relate to 2014. After the first MDM, 274 patients (23% of total referrals) were identified as having severe SDB, and were referred directly for a CPAP trial. As shown in Fig. 4, median waiting time from referral to CPAP initiation for ‘high risk’ patients (driving risk, heavy goods vehicle drivers etc.) identified as having severe OSA by the first MDM was 48 days. Waiting time from referral to CPAP initiation for other patients with severe SDB identified at the first MDM was 261 days.

Forty-eight percent of patients obtained a definitive answer from the first MDM—either that they had SDB severe enough to qualify for hospital-funded CPAP (23%), or that they should be managed by their GP (25%), the latter because: (1) they did not have a significant sleep disorder; or (2) that their sleep disorder was not severe enough for funded treatment, or (3) it was deemed appropriate to be managed in a primary care setting with Community HealthPathways advice and support.

Forty-nine percent of patients required a more complex sleep study after the first MDM. The majority of these studies were home-based, either flow-based Level IV or Level III studies.^14^ Only 8% of total referred patients were deemed to require Level II or I polysomnographic studies, following the first MDM.

A small number of assessments (34, 3% of total) were deemed by the MDM to ‘require further information’ prior to making a decision. This category included patients requiring spirometry and arterial blood gas analysis, or where further information was requested from the assessing practice.

After the second study and MDM, a further 151 patients were shown to have SDB severe enough to qualify for funded CPAP therapy. The median time from referral to second MDM was 208 days. Thirty of these 151 patients were assessed subsequently as requiring urgent CPAP provision. In addition, a further 337 patients were deemed appropriate for GP managed care. A total of 432 patients (37% of total referrals) were identified as having SDB severe enough to qualify for hospital-funded CPAP by this pathway, while 657 patients (56%) were sent back to be managed by their GP. Of these latter patients, 38% had an oxygen desaturation index (ODI) of less than 5.0, i.e., no significant SDB. The median ODI of the other 62% of patients defined by the MDM as having mild or moderate SDB was 11.0 (range 5.0–32.0).

Over the total assessment process, 122 patients (10.4% of total) underwent a polysomnographic Level I or II study, while 51 patients (4% of total) needed to see a sleep specialist for face-to-face assessment to identify complex sleep-related issues, and to determine the need for further testing or treatment. Thirty-five patients (3% of total) required three or more studies as part of the MDM process, mainly for requirement of a more detailed sleep study.

## Discussion

### Main findings

This paper describes the establishment of a new, predominantly community-based sleep assessment service, delivered by approved general practices in partnership with and supported by a hospital based MDM, sleep service, and sleep specialists. This service is now delivering ~1200 sleep assessments per year, compared to ~400 per year in the previous system. Waiting time to assessment and treatment has markedly reduced, with very rapid identification and treatment of severe SDB in ‘at-risk’ patients such as commercial drivers.

### Strengths and limitations of this study

A number of factors have been critical to this process. This service was developed as part of a locally driven health service philosophy known as the Canterbury Initiative, which facilitates and encourages provision of integrated services based in community settings. Other examples of integrated services in Canterbury include community spirometry provision,^15^ skin lesion excision,^16^ and pipelle biopsies.^17^ Specific factors critical to this current service include hospital clinician and sleep unit support, especially around interpretation of sleep studies, coordination of the MDM process, and quality assurance. In addition, the service is supported by a comprehensive training program, appropriate funding, a clear governance structure, and close integration between primary and secondary care clinicians and the sleep unit.

The majority of MDM decisions could be undertaken using the standardised community assessment and simple home-based sleep studies. Only a small proportion of patients required polysomnography and/or sleep specialist assessment prior to a management decision being made.

In 2014, 159 patients subsequently offered CPAP for SDB (37% of the patients identified as qualifying for hospital funded CPAP) were not initially identified using oximetry, but required a more complex sleep study. This is consistent with previous studies of the sensitivity of oximetry in the diagnosis of severe SDB.^18^ The requirement for a second test, and subsequent discussion in the MDM, increased wait-time to a decision for CPAP. However, once severe SDB was diagnosed, especially in ‘at risk’ patients, time to CPAP initiation was appropriately short. In 2014, a total of 30 patients out of the total of 1173 tested were only identified as being at ‘high risk’ from SDB following the second MDM.

### Implications for future research, policy and practice

Over the period of establishment of the service, the overall severity of SDB identified has not decreased (data not shown), indicating ongoing considerable previously undiagnosed severe SDB.

In 56% of cases, it was deemed that general practice management was the appropriate outcome following assessment. Sixty-two percent of these assessments were identified as having at least some degree of SDB (median ODI 11.0), but which would not qualify for hospital-funded treatment using our local DHB criteria. In New Zealand, criteria for publically funded provision of CPAP therapy vary from region to region, with most large centres only funded to provide CPAP for patients with severe OSA, or those with more mild disease but where there are other important factors, for example, very sleepy and/or occupational drivers. While this process has identified a significant number of patients with mild-moderate OSA, which cannot be managed by our service, it does provide the patients’ GP with a diagnosis, and the opportunity to explore other management options such as lifestyle modification, or self-funded CPAP therapy.

An earlier audit of this process (Lines, R., Epton, M., Kelly, P. T., Powell, S. A. and Hlavac, M. ‘Outcomes of patients returned to general practitioner coordinated care after assessment via a sleep disorders clinical pathway’. (Personal communication 2012)). was undertaken to identify the outcomes of patients returned to their GP. This audit highlighted the importance of sending copies of MDM outcomes to the patient as well as the GP, with the suggestion that the patient makes an appointment to discuss the results with their GP, and review community-based options for management of OSA.

While it was originally hoped that the overall workload of the sleep unit might be reduced by this pathway, in reality workload has increased due to the greater need for more complex sleep studies, as well as the need to coordinate the MDM and undertake administrative activities around the assessment process. This is due to the system recognising and managing previous unmet need. It does allow the most appropriate assessment to be undertaken in a more timely fashion. The establishment of this pathway has, however, not led to devolution of all sleep assessments into community settings.

The sleep pathway has increased work for approved provider practices and required them to provide a comprehensive initial assessment for patients previously referred to and managed by specialist services. Most approved provider practices have welcomed this change as increasing their professional capacity, business size, and knowledge of SDB. In general, the overall workload for primary care related to SDB has also increased, due to previous unmet need being uncovered by a new service. This has also required general practices to increase their knowledge to manage patients with mild and moderate SDB, and other common sleep disorders such as restless legs, unable to be managed by specialist services. In Canterbury, many other services have been partially devolved to the community, with flow onto specialist waiting lists being strictly managed.^19^

One benefit of this pathway is that the patient and their primary care team are receiving clarity about the diagnosis of conditions such as mild and moderate SDB at a much earlier stage than previously. This allows decisions to be made in primary care about future treatments that would not otherwise be funded by the public system.

Though the median time from referral to assessment was satisfactory, there were a number of patients whose assessment was delayed, sometimes for a number of months. The majority of cases related to administrative process at a practice level, in addition to difficulties making appointments with some patients in a timely fashion. We are addressing this issue with an electronic assessment process, with tighter service specifications for times to assessment. These issues are the subject of ongoing audit.

## Conclusions

Community assessment for SDB using this service model leads to increased number of assessments, reduced waiting times both for assessments and treatment, especially in patients identified as having ‘high risk’ severe SDB. Few patients assessed using this pathway required polysomnography and/or sleep physician assessment.

## Methods

### Referral criteria

Community-based sleep assessment was funded by the Canterbury DHB for patients older than 15 years with suspected OSA. Investigation and treatment of insomnia was not funded by this pathway. Children requiring sleep assessment were referred to the Christchurch Hospital Paediatric Service. Patients with high suspicion of complex sleep disorders such as narcolepsy or parasomnia were referred directly to the hospital sleep specialist service.

### Details of the assessment tool

In 2007, a comprehensive community sleep assessment tool, using a number of validated components, was developed by a sleep clinical nurse specialist (SP) with support from sleep physicians and the sleep service. A copy of this questionnaire is available from the authors on request. This included a detailed sleep history, including duration and sleep fragmentation questions; questions relating to snoring and apnoea, restless legs/periodic limb movements of sleep, and parasomnia; caffeine, alcohol and drug history; weight and weight gain; driving and work-related incidents; and upper airway surgical history. A focused examination was also performed of the nose, mouth, jaw and upper airway, including the Friedman classification of the oropharynx and tonsils.^20^ In addition, the Berlin questionnaire^21^ was used to identify patients with a high pre-test probability of OSA, and the Epworth Sleepiness score^22^ was recorded. A Level IV sleep study (overnight oximetry) was undertaken in conjunction with this assessment—see below for details.

The community sleep assessment tool was delivered by trained and accredited general practice-based teams (Approved Providers) to an agreed service specification. For further details of training and service specifications, see below. In areas where a local practice could not provide the service, trained mobile nurses were employed to deliver sleep assessment services in a local practice.

Individual practices collated the results of the clinical assessments and Level IV sleep studies, and forwarded these to a central processing point for invoicing and quality control. The assessments were then sent to the sleep MDM for clinical decision making (see below). At all points in the assessment process there are agreed processes for community clinicians to alert the MDM and sleep unit about the presence of severe disease, including driving or occupational risk, to allow more rapid processing and action.

### Level IV sleep study—overnight oximetry

A range of oximeters were used by practices, including NONIN/Respironics 8500 M, NONIN Palmstat (Nonin Medical Inc., Plymouth, MN,USA), Bitmos 901 and Bitmos 801 (Bitmos GmbH, Düsseldorf, Germany). Standardised diagnostic software, Nvision (Nonin Medical Inc.) and Satview (Bitmos GMbH) was selected for use by the service. A single night study was undertaken for each patient. Data was downloaded and analysed by general practice staff, including quality assessment. Oximetry was graded for quality based on the length of study and signal integrity. Oximetry specifications and scoring criteria were in line with American academy of sleep medicine guidelines.^23^ An acceptable study contained a minimum of 4 h of data.

### Sleep MDM and subsequent testing and treatment

All assessments were discussed at a combined sleep MDM. These meetings occurred weekly, or more often if circumstances dictated. The sleep MDM consisted of senior sleep physicians and trainees, sleep clinical nurse specialists, community respiratory nurses, with community assessors regularly attending. Given the complexity around assessment of sleep disorders, and the importance of determining appropriate treatment or the need for further more complex testing, it was felt mandatory that all cases be assessed in this way. Assessments were categorised into four broad groups:

1. *Confirmed OSA*. Patients identified as having OSA severe enough to qualify for publically funded treatment were offered CPAP via the hospital sleep service (see funding criteria in Table 1). Patients with confirmed OSA who did not meet public funding criteria for CPAP were referred back to their referring doctor with details of how to access privately funded CPAP or other treatment modalities such as oral appliances. In addition, advice about lifestyle measures such as weight loss and exercise were provided to referring doctors, via Community HealthPathways information pages.

**Table 1:** Criteria for funded CPAP therapy in Canterbury

Criteria for funded CPAP therapy
• Severe sleep apnoea (AHI or ODI > 30), or
• Evidence of mild/moderate sleep apnoea in combination with one or more of the following:
Severe subjective daytime hypersomnolence (ESS > 17).
Evidence of mild/moderate sleep apnoea in combination with an occupational risk.
Evidence of mild/moderate sleep apnoea with significant co-morbidities.

*AHI*, apnoea hyponoea index, *ESS*, Epworth sleepiness scale
2. *Patients requiring more complex sleep studies*. Patients identified as requiring more complex sleep studies to confirm OSA, due to high clinical suspicion and an inconclusive Level IV study, or patients with other sleep disorders requiring further investigation, were brought forward for more complex studies from the sleep unit. The appropriate sleep study was determined by the MDM, using the information from the community assessment. More complex studies included home-based studies such as Level III and Level II studies, or hospital-based diagnostic polysomnography (Level I) and multiple sleep latency testing. Following these more complex studies, patients were re-discussed at the sleep MDM.
3. *Patients requiring sleep physician assessment*. Where the diagnosis was not clear from the initial assessment, a face-to-face assessment by a sleep specialist was undertaken to determine either the likely diagnosis or the need for further testing.
4. *Patients with no concerning sleep disorder or very mild disease*. This category includes patients with insomnia. Advice for management of these patients was given to the referring general practitioner, based on Community HealthPathways information.

### Details of the training programme

General practitioners and practice nurses from approved provider practices, and community respiratory nurses were required to attend a training course coordinated by the integrated sleep service. This education session consisted of three components: (1) overview of SDB, (2) pulse oximetry, and (3) clinical assessment tools for SDB. At the completion of the session the participants were provided with an information booklet. The practice nurses who completed the training and performed ten assessments to the quality determined by the integrated sleep service became competent approved assessors. The assessors attended a 1-day refresher course 12 months later and participated in refresher courses every 2 years. For accreditation purposes, a record of competencies was kept by the integrated sleep service.

Doctors from approved provider practices also attended education sessions in test reporting and interpretation of the sleep assessment recordings. These sessions were coordinated by the sleep unit and respiratory physicians. All community assessors were encouraged to attend the MDM sessions. Quality assurance feedback to community assessors was provided by sleep clinical nurse specialists, and sleep physicians, as required.

### Governance, rollout and funding

Targeted general practices were given the opportunity to become a contracted primary care provider for this testing. If possible, these practices were to be located in different parts of urban Christchurch and rural Canterbury. In the contract, the responsibility for referral management and testing remained with the approved provider practice, with the integrated sleep service providing support, guidance and quality assurance. The providers were paid a subsidy for each completed assessment.

Funding for the service was provided by the Canterbury DHB. Training of practice nurses was funded by the individual practices, who also purchased the oximeters. An integrated sleep service governance group was convened to monitor the service, including service specifications and performance indicators.

A community respiratory physician was appointed, with one of their responsibilities to oversee the sleep assessment service and provide medical and sleep assessment interpretation guidance to approved provider practices. The integrated sleep service and the community respiratory physician were jointly responsible for monitoring the quality of the sleep assessment tests and interpretation of the results.
